# Reexamination of Human Rabies Case with Long Incubation, Australia

**DOI:** 10.3201/eid1412.080944

**Published:** 2008-12

**Authors:** Nicholas Johnson, Anthony Fooks, Kenneth McColl

**Affiliations:** World Health Organization Collaborating Centre for Rabies and Rabies-Related Viruses, Weybridge, UK (N. Johnson, A. Fooks); Australian Animal Health Laboratory, Geelong, Victoria, Australia (K. McColl)

**Keywords:** rabies incubation period, Australia, letter

**To the Editor:** Long incubation periods are an occasional feature of infection with rabies virus and should be considered in human cases of acute encephalitis in rabies-free countries where there has been a history of travel to rabies-endemic areas (*1*). Until 1987 Australia had recorded only 1 case of travel-acquired rabies. However, in 1990 an extreme case of long-incubation rabies was diagnosed. The patient was a 10-year-old girl of Vietnamese origin in whom rabies developed after she had lived continuously in Australia for almost 5 years (*2*). A thorough investigation of the case history by public health officers concluded that the likely source of infection was Vietnam, which the girl left in July 1984, suggesting an incubation period >6.5 years. Preliminary sequence analysis of a fragment of the rabies virus genome extracted from postmortem samples taken from the patient confirmed that the likely origin was Southeast Asia (*3*).

Since that time, 2 major developments occurred that suggested that the case should be reexamined. The first was the discovery of a bat lyssavirus in Australia (*4*). In addition to being closely related to rabies virus, Australian bat lyssavirus (ABLV) had been isolated from 2 patients with fatal cases in Australia, one of whom was deduced to have had a potential incubation period of 27 months (*5*). The second development was the increase in phylogenetic investigations of rabies viruses in many countries across Asia including Sri Lanka, the Philippines, India, People’s Republic of China (*6*), Indonesia, and, importantly, Vietnam (*7*).

After the patient’s death, a decomposed sample of the patient’s brain was sent to the Australian Animal Health Laboratory at Geelong. Although live virus was not isolated from this sample, several short sequences of the viral genome were amplified by PCR. From one of these, a 200-bp sequence of the nucleoprotein gene was derived that confirmed the clinical diagnosis of rabies. This sequence was compared to a small panel of virus sequences known at the time. Although limited, with no representative sequences from Vietnam, this early comparison indicated that the virus was of Asian origin (*3*).

To reinvestigate this case, we reassessed one of the other virus sequences originally generated from this case with a panel of rabies virus sequences from Southeast Asia, including viruses from Vietnam, China, and examples of ABLV. The original DNA fragment that yielded this sequence was also amplified from postmortem brain samples and the 306-bp sequence (GenBank accession no. EU854576) corresponded to positions 71–376 of the Pasteur virus genome (NC_001542) encoding the first 102 aa of the viral nucleoprotein. Further sequences were obtained from published studies submitted to GenBank. Sequences were aligned and edited with DNAstar Lasergene version 7 suite of programs (DNAstar Inc, Madison, WI, USA). Neighbor-joining and maximum likelihood analysis were undertaken by using PHYLIP version 3.5 as previously described (*8*). A sequence derived from a human case of European bat lyssavirus type 2 (EF157977) was used as the outgroup. One thousand replicates were used to assess bootstrap support for the phylogenic analysis.

The phylogenetic tree produced by this investigation indicates that the 1990 sequence falls within the rabies virus clade and is clearly distinct from the 4ABLV sequences included (Figure). These findings exclude the possibility that the young Vietnamese girl was infected with ABLV, now known to be endemic within Australian bats, after she arrived in Australia. The virus from the patient belonged within the rabies virus clade, forming a separate lineage most closely related to a subgroup of viruses found in China (*6*). The sequence is distinct from a small number of sequences derived from rabies viruses in Vietnam, which suggests that China is a stronger candidate for the source of the virus than her native country.

**Figure:**
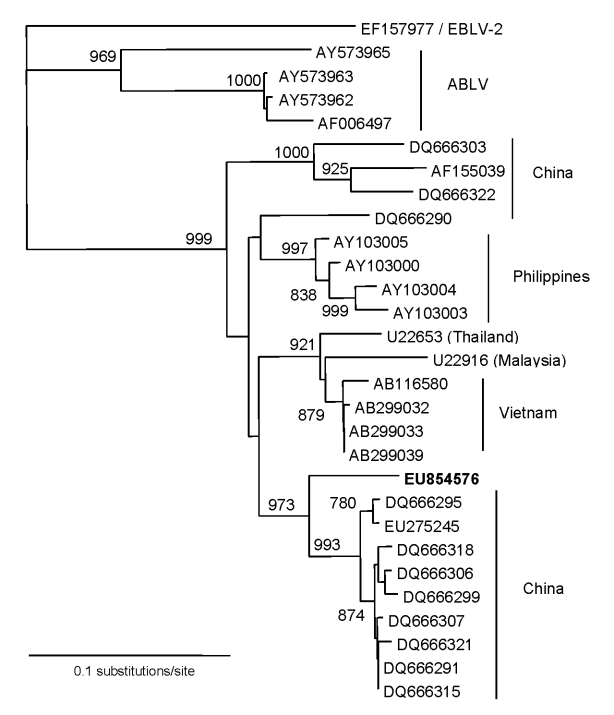
Phylogenetic analysis of lyssavirus nucleoprotein sequences (306 bp) derived from viruses of Asian origin. European bat lyssavirus type 2 has been used as the outgroup. The human sequence is shown in **boldface**.

Although the case history could not provide evidence for interaction with a dog while her family was in Hong Kong Special Administrative Region, rabies was endemic within the colony at the time that the patient’s family was resident. From 1980 through 1984, 5 human cases were recorded (*9*). Only 2 case-patients had clear evidence of a dog bite; histories for the remaining 3 cases provided no evidence for an animal bite. From 1956 to 1979, Hong Kong had been free of rabies, but the disease had reentered the colony shortly after its incidence had increased in the neighboring Chinese province of Guangdong. If Hong Kong was where the young girl was infected, it would indicate an incubation period of 4.5–6 years.

Such long incubation periods are rare for rabies virus infections. An earlier epidemiologic study of 177 cases in Amritsar, India, demonstrated that rabies developed within 6 months of exposure in 90% of human cases (*10*). However, the thorough documentation of a small number of cases (*1*,*2*) suggests that clinicians need to be aware of the importance of including travel history over several years in cases of unexplained encephalitis.
